# Reconciliation-based detection of co-evolving gene families

**DOI:** 10.1186/1471-2105-14-332

**Published:** 2013-11-20

**Authors:** Yao-ban Chan, Vincent Ranwez, Celine Scornavacca

**Affiliations:** 1 ISEM, Université Montpellier 2, Montpellier, 34095, France; 2 Montpellier SupAgro, UMR AGAP, F-34060 Montpellier, France; 3 Institut de Biologie Computationnelle (IBC), 95 rue de la Galéra, 34095 Montpellier, France

## Abstract

**Background:**

Genes located in the same chromosome region share common evolutionary events more often than other genes (e.g. a segmental duplication of this region). Their evolution may also be related if they are involved in the same protein complex or biological process. Identifying co-evolving genes can thus shed light on ancestral genome structures and functional gene interactions.

**Results:**

We devise a simple, fast and accurate probability method based on species tree-gene tree reconciliations to detect when two gene families have co-evolved. Our method observes the number and location of predicted macro-evolutionary events, and estimates the probability of having the observed number of common events by chance.

**Conclusions:**

Simulation studies confirm that our method effectively identifies co-evolving families. This opens numerous perspectives on genome-scale analysis where this method could be used to pinpoint co-evolving gene families and thus help to unravel ancestral genome arrangements or undocumented gene interactions.

## Background

Species from the same ecosystem may share common environmental factors (e.g. related to the local climate or to the arrival of new species in the ecosystem) or be interdependent, and their evolution can be related. In the vast majority of cases, the footprint of this dependence is minimal, but in some cases, such as predator-prey, host-parasite or symbiotic relationships, species influence each other so much that their co-evolution can be detected [1-3]. Similarly, nucleotides and amino acids that are located close to one another on the genome share common local factors (e.g. specific nucleotide composition bias or underlying mutation rates due to the functional importance of the locus) and influence each other (e.g. because they are in the same codon, part of the same active site of a protein or because one is part of a transcription factor controlling the transcription level of the other).

The problem of detecting co-evolution at the amino acid level has been extensively studied recently ([4,5]; among others). However, at a broader level, neighbouring genes can also co-evolve, sharing common evolutionary events such as segmental duplications [6] and local evolutionary factors such as the proximity of recombination hotspots or centromeres [7]. Protein interactions, e.g. being part of the same protein complex or biological pathway, can also induce co-evolution at the gene level. Relatively little work has been done on detecting co-evolution at the gene level [8-12].

To detect gene co-evolution, one has to observe it in a significant number of species. As more and more full genomes/transcriptomes are sequenced, more raw data needed to detect co-evolving genes becomes available. Being able to accurately detect co-evolving genes would, among other things, help to (a) pinpoint possible functional interdependence, allowing us to annotate genomes from non-model species; (b) infer ancestral proximity among genes, allowing us to reconstruct ancestral genome arrangements [11]; or (c) cluster genes to reconstruct the Tree of Life in a divide-and-conquer framework [13,14].

In [12], Cohen *et al.* proposed a probabilistic method to detect co-evolutionary interactions from phylogenetic profiles, using gain and loss events. They used their method to study a group of 4593 prokaryotic gene families and construct a co-evolution network. This yielded several clusters of genes which corresponded to identifiable functional pathways.

In this paper, we propose a novel probabilistic method to detect co-evolution. Our method differs from that of [12] in that it is based on species tree-gene tree reconciliations. Reconciliation methods construct a mapping between a gene tree and a species tree to explain their incongruence by macro-evolutionary events such as speciations, gene duplications, horizontal gene transfers etc. Several reconciliation methods have recently been developed following parsimonious or probabilistic paradigms (see [15] for a review). By using reconciliations, we are able to distinguish between different types of events and take into account uncertainties on such events [16,17].

Our method has advantages over that of [12] in that (a) it can measure co-evolution between genes with small or different numbers of events; (b) it can take into account several possible evolutionary scenarios for each gene, reflecting inference uncertainties; and (c) it uses a theoretical model-based framework to compute *p*-values for the co-evolution score, rather than bootstrapped simulations as done in [12]. Simulations show that our method is effective in detecting co-evolution between genes, even when it is relatively weak. It is also time-efficient, which allows us to conduct genome scale analysis to search for undocumented co-evolution among thousands of gene families.

## Preliminaries

Let *T* = (*V*(*T*), *E*(*T*)) be a (rooted) tree with labelled leaf vertices. We denote the leaves of *T* by *L*(*T*) and the (multi)set of all labels of those leaves by L(T). Given a vertex *x* ∈ *V*(*T*), we denote by *x*_*p*_ its parent and by *y* ≤ *x* the fact that a vertex *y* is a descendant of *x*.

We define a gene tree *G* as a tree where each leaf represents an extant gene. Likewise, we define a species tree *S* as a tree in which each leaf represents a distinct extant species. The labels of the leaves of *S* are unique since they are the identifiers of these species. In gene trees, internal vertices may represent various evolutionary events (e.g. speciation, duplication), while in the species tree they all represent speciation events. In this paper, we suppose that gene and species trees are rooted and binary. Finally, we assume that the genes of *G* come from the genomes of species present in *S*, in particular that each label of L(G) appears in L(S) (denoted by L(G)⊑L(S)).

A species tree *S* is said to be *dated* if it is associated to a function *θ*_*S*_ which represents the time separating a vertex from the current time, i.e. $\theta_{S}:V(S)\to R^{+}$ such that if *y* ≤ *x* then *θ*_*S*_(*y*) ≤ *θ*_*S*_(*x*) and if *x* ∈ *L*(*S*) then *θ*_*S*_(*x*) = 0. Using a *subdivision* of *S* rather than *S* itself when computing reconciliations has been proven to ensure time-consistency of gene transfers in polynomial time [18]. The subdivision *S*^′^ of *S* together with an associated time function $\theta_{S^{\prime }}$ is constructed as follows: firstly, for each node *x* ∈ *V*(*S*) ∖ *L*(*S*) and each edge (*y*_*p*_, *y*) ∈ *E*(*S*) s.t. $\theta_{S}(y_{p})>\theta_{S}(x)>\theta_{S}(y)$, an *artificial* node *w* is inserted along the edge (*y*_*p*_, *y*), with $\theta_{S^{\prime }}(w)=\theta_{S}(x)$; secondly, for nodes *x* ∈ *V*(*S*^′^) corresponding to nodes already present in *S*, we set $\theta_{S^{\prime }}(x)=\theta_{S}(x)$.

In this paper, we use the combinatorial reconciliation model of Doyon *et al.*[18], called the DTL model. We refer the reader to this paper for a formal definition of reconciliations. This model considers (as possible macro-events that shape the genome) speciations, duplications, transfers and losses of genes. For algorithmic reasons losses are never considered alone, so the atomic events of this model are: a speciation (), a duplication (), a transfer (), a transfer followed immediately by the loss of the non-transferred child (TL), a speciation followed by the loss of one of the two resulting children (SL), a no event () that only reflects the fact that a gene lineage has crossed a time boundary, and a contemporary event () that associates an extant gene to its corresponding species.

The method of [18] calculates the most parsimonious reconciliation under this model. However, there often exist several most parsimonious reconciliations. Those reconciliations constitute what we call a reconciliation space, which can be efficiently stored in the reconciliation graph introduced by Scornavacca *et al.*[16].

## Methods

In this section we present our new methodology to detect whether or not two gene familes have co-evolved. We take as input two gene trees *G*_1_ and *G*_2_ and a dated tree *S* such that $L(G_{1})⊑L(S)$ and $L(G_{2})⊑L(S)$.

Our co-evolution detection method consists of three main steps: 

1. We reconcile each of the two gene trees to $S^{\prime }$ (the subdivision of *S*) to produce two corresponding reconciliation spaces. Event sets are then extracted from these two spaces. Details are given in the “Computing the weighted event sets” section.

2. We calculate a *co-evolution score* which quantifies the similarity between the two event sets. Details are given in the “Computing the co-evolution score” section.

3. We calculate the *p*-value of the calculated score under a model of independent evolution. If this *p*-value is less than an appropriate threshold (reflecting the acceptable error rate for false positive co-evolution detection) we consider that *G*_1_ and *G*_2_ co-evolved. Details are given in the “Computing the *p*-value” section.

### Computing the weighted event sets

We use the method of [16] to reconcile each of the two gene trees to the subdivided species tree, using equal costs for ,  and  events. This yields two reconciliation spaces *RC*_1_ and *RC*_2_ which contain all of the most parsimonious reconciliations between *G*_1_ (respectively *G*_2_) and *S*. By taking the multiple reconciliations of *RC*_1_ and *RC*_2_ into account, we can explore a broad set of possible events, assess their reliability and remove the danger of artifacts arising in a single reconciliation.

Each reconciliation, according to the DTL model, yields a set of events with types from {S,D,T,TL,SL,∅,C}. However,  and  events are determined by the species tree, and  events are artifacts due to the use of subdivision. Therefore, coincident events of these types are not an indication of co-evolution, and we discard them. Likewise, we consider SL events only as  events. Furthermore, TL events are considered as two separate  and  events.

We are now left with only ,  and  events which we extract from the reconciliation spaces. These events are characterised by their type and their position in the considered gene and species trees. Here, we “undo” the subdivision and consider the position of the event in the original species tree *S* rather than the subdivided tree $S^{\prime }$.

For each branch *b* ∈ *E*(*S*), gene *u* ∈ *V*(*G*_1_) and event type E∈{D,T,L}, we define $w_{1}{\left(b,u\right)}_{E}$ to be the fraction of reconciliations of *RC*_1_ in which *u* is mapped to an event of type  on branch *b*. Note that this means that transfers departing from the same branch of *S* but reaching different branches are considered identical, for simplicity (otherwise there are too many possible transfers to be time-efficient in later computation). Then we define the set

$$W_{1}{\left(b\right)}_{E}=\underset{u\in V(G)}{⋃}\{w_{1}{\left(b,u\right)}_{E}\},$$

which contains the weights of all events of type  on branch *b*.

Since the frequency of an event over most parsimonious reconciliations has been shown to be a good indicator of its reliability [17], we use $w_{1}{\left(b,u\right)}_{E}$ as an estimate of the probability that this event has really occurred in *G*_1_. This provides us with a set of possible events together with their probabilities according to *G*_1_. Another set is obtained from *RC*_2_ in a similar way.

Note that these weighted event sets can be obtained from any reconciliation method, for example by taking into account the set of Near-optimal Parsimonious Reconciliations (NPRs, see [17]), rather than focusing only on most parsimonious reconciliations. Having a set of reconciliations is preferable, since it reflects the inherent uncertainty of reconciliation inference and event prediction. It also allows us to have probability values associated to each event, whereas a single reconciliation only has the presence or absence of events. If only given a single reconciliation, one can also obtain a set of associated (sub-)optimal reconciliations, e.g. reconciliations that are reachable by a small number of the operators described in (Chan, Ranwez, Scornavacca: Exploring the space of gene/species reconciliations with transfers. Submitted to *J Math Biol*).

In fact, we use reconciliations only as a tool to produce the weighted event sets, which are the input to the remainder of the method. In theory, any method which produces a weighted set of genetic events (even if they are not DTL events) can replace this step. We use reconciliations because they provide a straightforward way to calculate the event sets, and there are already efficient algorithms for computing the reconciliation spaces.

### Computing the co-evolution score

Events of the same type which occur at approximately the same time in both *G*_1_ and *G*_2_ support a hypothesis of co-evolution. Therefore, we calculate a statistic which measures the amount of co-evolution based on the number of such events which are inferred from the reconciliations.

Given two reconciliations — one for *G*_1_ and one for *G*_2_ — we could define the *co-evolution score* to be the number of D,T or  events which occur in both reconciliations on the same branch.

However, since we have computed a set of weighted events for each gene resulting from several reconciliations, the co-evolution score between *G*_1_ and *G*_2_ is computed as follows: 

1. We consider the weight associated to each event $w_{1}{\left(b,u\right)}_{E}$ to be the probability that this event has occurred in *G*_1_. We make the (strong) assumption that any such event is independent from any other event represented by $w_{i}{\left(b^{\prime },u^{\prime }\right)}_{E^{\prime }}$ for *i* = 1, 2.

2. For all branches *b* ∈ *E*(*S*) and element types , we calculate the probability of having 0, 1, …, *n* events of type  on *b*, where $n=|W_{1}{\left(b\right)}_{E}|$. This is done via recursion as follows: suppose $W_{1}{\left(b\right)}_{E}=\{p_{1},\ldots ,p_{n}\}$. Let *X*_*i*_ be a variable representing the number of actual events from the first *i* possible events represented in this set. Then for *i* = 1, …, *n* and *x* = 0, …, *i*, we have

$$P(X_{i}=x)=p_{i}P(X_{i-1}=x-1)+(1-p_{i})P(X_{i-1}=x),$$

where the initial conditions are *P*(*X*_*i*_ = - 1) = 0 and *P*(*X*_0_ = *x*) = *I*(*x* = 0).

3. We do the same for *G*_2_, using the notations *Y* and *m* instead of *X* and *n*. The variables *X*_*n*_ and *Y*_*m*_ represent the total number of actual events of type  on this branch.

4. For all branches *b* of *S* and element types , we compute the expected number of events in common:

$$\begin{array}{l} E(\text{number of events in common})\\ \;=\overset{n}{\underset{x=0}{\sum }}\overset{m}{\underset{y=0}{\sum }}\min(x,y)P(X_{n}=x)P(Y_{m}=y). \end{array}$$

We define the co-evolution score between *G*_1_ and *G*_2_ given *S* as the sum of this value over all branches of *S* and event types.

As an example, suppose that for a particular branch *b* ∈ *E*(*S*), we have $W_{1}{\left(b\right)}_{D}=\{1,0.5,0.5\}$ and $W_{2}{\left(b\right)}_{D}=\{0.6,0.2\}$. The distributions of *X*_3_ and *Y*_2_ for this combination (b,D) are calculated using the recursion formula above as detailed in Tables 1 and 2.

**Table 1:** Example probability calculation 1

***G***_**1**_	**0**	**1**	**2**	**3**
*X*_1_	0	1		
*X*_2_	0	0.5	0.5	
*X*_3_	0	0.25	0.5	0.25

Probabilities of having 0,1,2,3 duplications in *G*_1_ on a branch *b* ∈ *E*(*S*) with $W_{\text{1}}{\text{(b)}}_{D}\text{=\{1, 0.5, 0.5\}}$.

**Table 2:** Example probability calculation 2

***G***_**2**_	**0**	**1**	**2**
*Y*_1_	0.4	0.6	
*Y*_2_	0.32	0.56	0.12

Probabilities of having 0,1,2 duplications in *G*_2_ on a branch *b* ∈ *E*(*S*) with $W_{\text{2}}{\text{(b)}}_{D}\text{= \{0.6,0.2\}}$.

The contribution of (b,D) to the co-evolution score is

$$\begin{array}{lll} \;\text{contribution}(b,D) & =0\;(0\times 0.32+0\times 0.56+0\times 0.12\; & \;\\ & \;+0.25\times 0.32+0.5\times 0.32+0.25\; & \;\\ & \;\times 0.32)+1(0.25\times 0.56+0.25\; & \;\\ & \;\times \;0.12+0.5\times 0.56+0.25\times 0.56)\; & \;\\ & \;+2(0.5\times 0.12+0.25\times 0.12)\; & \;\\ & =0.77.\; & \; \end{array}$$

### Computing the *p*-value

The co-evolution score measures the dependence between two gene trees given a species tree. However, its distribution is highly dependent on the number of events in each reconciliation space. In order to assess the significance of the score, we compute the *p*-value associated to it.

To do so, we count the average number of events in each event set, which we denote (rounded up) by *N*_1_ and *N*_2_. For each branch *b* ∈ *E*(*S*) and event type , we call the combination (b,E) a *bin*, and denote by *B* the (arbitrarily) ordered vector containing all possible bins, over all branches *b* of the tree *S* and the 3 element types of . We denote the lengths (representing duration) of the respective branches in *S* by *l*_1_, …, *l*_*N*_, where *N* = 3|*E*(*S*)| is the number of bins. In this sequence, each branch length will occur 3 times, once for each event type.

We compute the *p*-value under a model that assumes that the genes do not co-evolve and all ,  and  events are distributed at random among the elements of *B*, with probabilities proportional to the branch lengths. Using a theoretical model allows us to efficiently calculate *p*-values without simulations which rely on bootstrapped data (as was done in [12]). This increases the reliability of the calculations and mitigates the influence of the independence assumption made when computing the co-evolution score (previous section, step 1 of the procedure).

#### 

**Definition 1. ***We define f*(*x*;*n*_1_, *n*_2_, *n*) *to be the probability that*, *if n*_1 _*and n*_2 _*events are randomly placed on the first n bins of B*, *there will be at least x events in common between the two event sets.*

Given a co-evolution score of *X*, our *p*-value is therefore *f*(*X*;*N*_1_, *N*_2_, *N*). We again calculate this statistic by recursion. Firstly, we define

$$\pi_{n}={\left(\overset{n}{\underset{i=1}{\sum }}l_{i}\right)}^{-1}l_{n}$$

to be the probability that an event is randomly assigned to bin *n* out of the first *n* bins, and

$$\text{BPr}(x;\pi ,n)=\left(\begin{array}{l} n\\ x \end{array}\right)\pi^{x}{\left(1-\pi \right)}^{n-x}$$

to be the binomial probability mass function with parameters *n* and *π*. Then we have the initial conditions

$$\begin{array}{lll} f(x;n_{1},n_{2},n)\; & =\;1\;\text{if}\;x\leq 0,\; & \;\\ f(x;n_{1},n_{2},n)\; & =\;0\;\text{if}\;x>\min(n_{1},n_{2}),\; & \;\\ f(x;n_{1},n_{2},1)\; & =\;I(x\leq \min(n_{1},n_{2})).\; & \; \end{array}$$

The recurrence is

(1) $$\begin{array}{ll} f(x;n_{1},n_{2},n) & =\overset{\min(n_{1},n_{2})}{\underset{i=0}{\sum }}\left[\text{BPr}(i;\pi_{n},n_{1})\text{BPr}(i;\pi_{n},n_{2})\right)\\ & \;\times \;f(x-i;n_{1}-i,n_{2}-i,n-1)\\ & \;+\overset{n_{1}}{\underset{j=i+1}{\sum }}\text{BPr}(j;\pi_{n},n_{1})\text{BPr}(i;\pi_{n},n_{2})\\ & \;\times f(x-i;n_{1}-j,n_{2}-i,n-1)\\ & \;+\overset{n_{2}}{\underset{j=i+1}{\sum }}\text{BPr}(i;\pi_{n},n_{1})\text{BPr}(j;\pi_{n},n_{2})\\ & \;\times \left(f(x-i;n_{1}-i,n_{2}-j,n-1)\right]. \end{array}$$

The variable *i* in the outside sum denotes the number of events in common between the two event sets in bin *n*. The first term considers the case where there are exactly *i* events in this bin in both sets. The second term accounts for the case where the first set has *j* > *i* events in this bin, but the second set only has *i* such events — the number of events in common is still *i*. The third term considers the mirrored version of the second term.

To calculate *f*(*X*;*N*_1_, *N*_2_, *N*), we calculate *f*(*x*;*n*_1_, *n*_2_, *n*) for all *x* ≤ *X*, *n*_1_ ≤ *N*_1_, *n*_2_ ≤ *N*_2_, *n* ≤ *N*, in order of increasing *n*. We can do this because (1) expresses *f*(*x*;*n*_1_, *n*_2_, *n*) in terms of *f* values where the fourth argument is *n* - 1 and the other arguments are not increased. The lower *f*(*X*;*N*_1_, *N*_2_, *N*) is, the stronger the evidence against the hypothesis that the genes did not co-evolve. To test the co-evolution hypothesis, we compare this number to a pre-defined threshold level, in general 0.05.

Note that the function *f* itself depends only on the species tree; only its arguments depend on the gene trees and co-evolution score. Because of this, we only have to perform the recursion once for every species tree, with the arguments set to the maximal values encountered in the set of genes. This allows us to quickly compute the values of the function for many genes which belong to the same species (which occurs, for example, in our simulations), and so process whole genome analysis to scan for undocumented gene family co-evolution.

## Results and discussion

In this section, we first describe the simulation protocol used to mimic gene family co-evolution along a species tree. We then provide and discuss the results obtained by our method on this dataset, which confirm its ability to detect when two gene families co-evolve.

### Gene tree simulation

We start with a dated species tree *S*. Every branch of *S* has an associated activity *a* — representing the overall rate at which // events occur on this branch — and specific rates for each individual event type $r_{D},r_{T},r_{L}$, with $a=r_{D}+r_{T}+r_{L}$. We simulate two gene trees simultaneously, with a parameter *c* ∈ [0, 1] (which we call the *co-evolution parameter*) representing the dependence between the two genes. Informally, an event in one gene tree has a probability *c* of also occurring in the other gene tree. For example, if *c* = 1 then the two trees must be identical, whereas if *c* = 0 they are completely independent.

To simulate the gene trees, we use a modified birth-and-death process which explicitly controls the co-evolution between the two genes. At the beginning of the process, the two genes are located at the root of *S* and *paired* (identified) to each other. At any time, the time *t*_*next*_ of the next // event in every existing unpaired gene is calculated by simulating an exponential variable with parameter equal to the activity of the branch (*x*, *y*) containing that gene. For gene pairs, this activity must be multiplied by a factor of $\frac{2}{1+c}$ for reasons that will be explained shortly. Then, if *t*_*next*_ ≤ *θ*_*S*_(*y*), the next event is determined to be a  event if *y* is a leaf, and an  event otherwise. If *t*_*next*_ > *θ*_*S*_(*y*), the next event is a // event and we rely on the relative rates $r_{D},r_{T},r_{L}$ to determine its type. If this event affects a gene pair, then: 

• If it is an , both genes in the pair must speciate. The left (respectively right) child of one resulting gene is then paired to the left (resp. right) child of the other.

• If it is a , the event will occur in one gene of the pair with probability 1, and in the other with probability *c*. If it occurs in both genes, the children are paired to each other as in the  case. If it occurs in only one gene, one of the resulting children is paired to the other gene (it does not matter which child).

• If it is a , we treat it the same as for a  event, with the added conditions that if it occurs in both genes, the transfer targets must be the same, and if it occurs in only one gene, the child which remains in the originating branch is paired to the other gene.

• If it is an , the event will occur in one gene of the pair with probability 1, and in the other with probability *c*. If it occurs in only one gene, the other gene is now unpaired.

It is now clear why the activity of a gene pair above is multiplied by $\frac{2}{1+c}$: each // event in a pair results in 1 + *c* actual gene events on average between the two trees. To achieve the correct marginal activity in each gene tree, we must multiply by the correcting factor.

**Figure 1:**
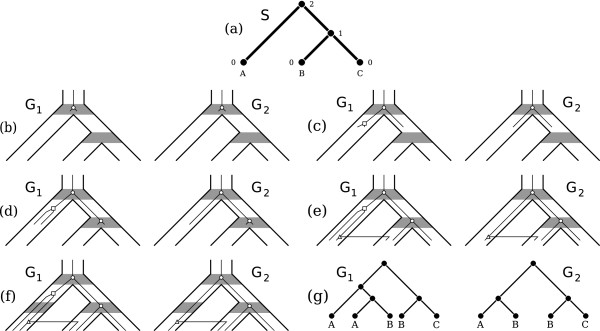
**Example simulation.** Example of simulating a pair of correlated gene trees, with 0 < *c* < 1. **(a)** The dated species tree. **(b)** The first speciation happens at date 2. **(c)** A duplication occurs at date 1.42. This duplication only occurs in the left gene tree; the right child of the duplication is paired to the original branch in the right gene tree. **(d)** Another speciation happens at date 1. **(e)** A transfer occurs in both trees at date 0.55. **(f)** There are no further events and we reach the time of the leaves (date 0). **(g)** The resulting gene trees.

We repeat this process until we reach the time of the extant species. This produces two (correlated) gene trees. An example of this process is given in Figure 1.

### Simulation results

We ran simulations using a phylogeny of 37 proteobacteria over a period of 500 million years as a species tree. We generated duplication, transfer and loss rates for each simulated gene independently, using the same scheme as [19]: the loss rate was randomly chosen in the interval [0.001, 0.0018], where the units are events per gene per million years; the ratio between the “birth” rate (sum of the duplication and transfer rates) and the loss rate was randomly chosen in the interval [0.5,1.1]; finally the proportion of the duplication rate to the birth rate was randomly chosen in the interval [0.7,1]. Both the species tree and the event rates were chosen in accordance with real dataset observations [20].

**Figure 2:**
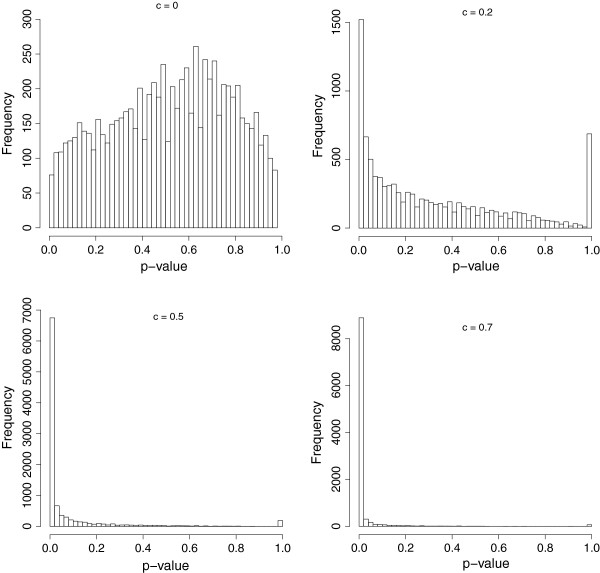
***p*-value distributions.** Sample distributions of the *p*-value for c = 0, 0.2, 0.5, 0.7.

We simulated 10 000 pairs of gene trees for each of the values of the co-evolution parameter *c* ∈ {0, 0.1, …, 1}. We then applied the procedure described in the “Methods” section to calculate the *p*-values for the co-evolution score. The results for *c*=0,0.2,0.5,0.7 are shown in Figure 2.

We observe that the *p*-value 1 is over-represented in all plots. This arises from the granularity of the simulations. More specifically, the *p*-value does not come from a continuous distribution, but from a variety of discrete distributions depending on *N*_1_ and *N*_2_, each with a moderate number of possible values. 1 is always one of these values (for when *X* = 0, i.e. there are no events in common), and so it is over-represented. This effect is more noticeable as *c* becomes smaller, because the likelihood of having no event in common grows larger.

It is apparent from Figure 2 that the *p*-value statistic is effective in distinguishing between co-evolving gene families and independent gene families. Even with quite low values of *c* such as 0.2, the distribution of the *p*-values is noticeably skewed towards 0. At higher levels of *c*, almost all the *p*-values are very close to 0.

If our underlying model is correct, then the case *c* = 0 in Figure 2 should have a uniform distribution. Even if we ignore the *p*-values of 1, our sample distribution is clearly not uniform (a *χ*^2^ goodness-of-fit test to a uniform distribution rejects this hypothesis with a *p*-value of less than 10^-15^). This is almost certainly due to the fact that our model assumptions are not an exact match for reality (or, indeed, our simulation protocol). However, the distribution is close enough to uniform that our assumptions appear to be reasonable. In fact the false positive rate for a threshold of 0.05 is only 0.024, less than expected under the underlying model.

**Figure 3:**
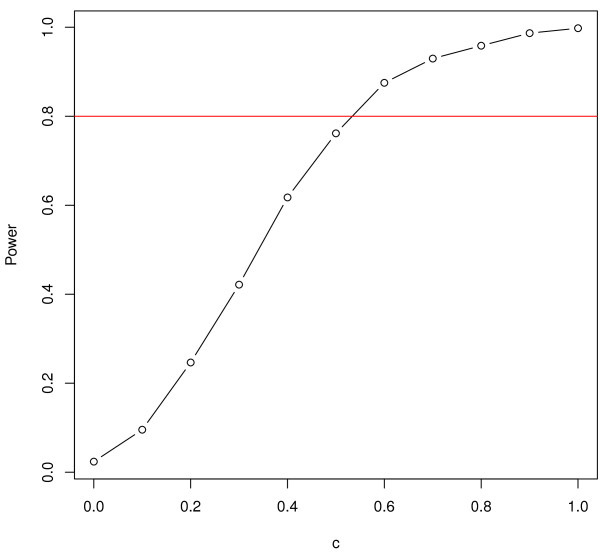
**Test power.** Power of the test for various values of *c*.

In Figure 3, we plot the power of the test (the true positive rate) for various values of the co-evolution parameter. As expected the power rises with *c*; it is greater than 0.8 (a standard threshold value for power measurement) for approximately *c*>0.52.

Further simulations (which we do not show the results of here) indicate that varying the event costs used in the reconciliation algorithm does not significantly impact these results.

### Comparison with the method of Cohen *et al.*

For a complete assessment of the effectiveness of our co-evolution detection algorithm, we compare it to the method of Cohen *et al.*[12] (henceforth referred to as Cohen’s method) on our simulated data.

We must stress that the two methods accept different input formats; while our algorithm takes gene trees as input, Cohen’s method only uses phyletic patterns of gene presence/absence in extant species, which can be extracted from the gene trees but do not contain all of their information. As such, we should expect our method to outperform Cohen’s method as it uses more information. On the other hand, the fact that our method requires more information as input is not a huge drawback, as full gene tree information is becoming more and more available in recent times.

We ran Cohen’s method on smaller test sets (1000 gene tree pairs) of simulated data for the co-evolution parameter values *c* = 0, 0.2, 0.5, 0.7; the smaller size was for efficiency reasons and is not expected to skew the results. Firstly, because Cohen’s method only compares genes with “exchangeability” (number of inferred gain/loss events) greater than some minimum value, only a small proportion (less than 15%) of the gene families were actually compared. Our method, which can compare any two gene trees, is clearly superior in this respect.

Even considering only those families which are compared by Cohen’s method, our method is still more sensitive. In Table 3 we show the proportion of gene tree pairs which were detected to have co-evolved, for each value of *c*. While we do have a slightly higher false positive rate, our method detects existing co-evolution more often for every value of *c*. We feel confident in asserting that if gene trees are available, our method performs better than Cohen’s method.

**Table 3:** **Comparison with the method of Cohen *et al.*** [12]

***c***	**Number of pairs compared by Cohen’s method (out of 1000)**	**Proportion of pairs with** ***p*-value < 0** ***.*** **05 (Cohen’s method)**	**Proportion of pairs with** ***p*-value < 0** ***.*** **05 (our method)**
0	68	0	0.024
0.2	80	0.08	0.247
0.5	133	0.56	0.762
0.7	144	0.92	0.930

## Conclusion

In this paper, we have devised an algorithm to detect and measure the strength of co-evolution between two gene families. It takes two gene trees as input, and uses their reconciliations to a common species tree to assess the co-evolution of the gene families. Simulation studies, and a comparison with the method of Cohen *et al.*[12], show that this test is an effective way of detecting co-evolution.

The detection of strong co-evolution among gene families can signal either a proximity or a functional relationship between the families. If working on a fully sequenced genome, the identification of co-evolution signals between distant genes could pinpoint ancestral genome rearrangements and/or strong functional links between those genes. If the genome is not fully sequenced, further study may be required to investigate the reason for co-evolution and to distinguish between proximity and functional relationships.

Further work includes the design of a clustering method based on co-evolution scores to provide biologists with clusters of co-evolving gene families rather than just pairwise co-evolution information. Another possible avenue for exploration includes extending the current method to include 3 or more gene families. We also plan, in collaboration with experts in bacterial evolution, to apply this method to the bacterial gene trees available in the HOGENOM database [21] to detect existing co-evolution among distant genes and to use this information to provide functional insights on un-annotated gene families.

## Competing interests

The authors declare that they have no competing interests.

## Authors’ contributions

The method was jointly devised by all the authors. YBC and CS programmed the method and ran the simulations. All authors wrote and revised the paper. All authors read and approved the final manuscript.
